# A Challenging Case of Recurrent Ogilvie Syndrome: Exploring Causes and Treatment Modalities

**DOI:** 10.1155/crgm/5378390

**Published:** 2024-12-14

**Authors:** Ahmad Alnasarat, Mostafa Elrazzaz, Nouraldeen Manasrah

**Affiliations:** ^1^College of Osteopathic Medicine, Michigan State University, Lansing, Michigan, USA; ^2^Department of Internal Medicine, Detroit Medical Center (Sinai Grace), Wayne State University, Detroit, Michigan, USA; ^3^Department of Cardiology, Medical College of Georgia, Augusta University, Augusta, Georgia, USA

## Abstract

**Introduction:** Acute colonic pseudo-obstruction (ACPO), or Ogilvie syndrome, is a rare condition marked by significant colon distention without mechanical obstruction. Symptoms include abdominal pain, bloating, nausea, vomiting, and an inability to pass gas or stool. Although common in males over 60, we report a challenging case of a 44-year-old man from Africa with recurrent abdominal distention and discomfort. Ultimately, he improved after receiving multiple treatment modalities, highlighting the complexities of Ogilvie syndrome management.

**Case Presentation:** A 44-year-old Nigerian male in the United States with hypertension and significant alcohol use disorder presented with altered mental status and bilateral lower extremity weakness after fasting and hydrochlorothiazide abuse. Initial diagnostics indicated metabolic encephalopathy from hypokalemia and dehydration. Despite aggressive treatment, he developed severe abdominal distension and obstipation. A CT scan showed diffuse colonic dilatation without a normal small bowel. Conservative measures failed, necessitating ICU transfer, TPN, and empiric antibiotics. Despite an initial response to colonoscopy decompression, the patient experienced recurrence. Neostigmine significantly improved his condition, leading to full recovery and discharge.

**Conclusion:** This challenging case highlights the complexities of managing Ogilvie syndrome and the importance of early identification and a stepwise approach to treatment. Incorporating a patient-centered plan utilizing conservative measures, pharmacological agents and endoscopic interventions are essential for improving outcomes in these cases.

## 1. Introduction

Acute colonic pseudo-obstruction (ACPO), known as Ogilvie syndrome, is characterized by significant colon distention that usually affects the cecum and right colon in the absence of mechanical obstruction [1]. It is initially described by Sir William Heneage Ogilvie in 1948 [2]. It is a rare condition affecting approximately 0.1% of adult inpatient admissions annually and poses a clinical challenge in practice [1]. Patients with Ogilvie syndrome may experience abdominal pain, bloating, nausea, vomiting, and an inability to pass gas or stool. Commonly identified risk factors include trauma, various medical illnesses, and cardiovascular disease. Additional predisposing factors encompass neoplastic conditions, renal dysfunction, respiratory failure, metabolic irregularities, and electrolyte imbalances, as well as the use of anticholinergic agents and opioids [3]. While it typically occurs in males above the age of 60, we report a challenging case of a 44-year-old male who had developed multiple predisposing conditions leading to recurrent episodes of Ogilvie syndrome during the hospital course. Addressing each potential factor along with providing more than one treatment method proved to be a clinical struggle.

A 44-year-old male, originally from Nigeria and residing in the United States since his teenage years, presented with a complex medical history marked by hypertension and a significant alcohol use disorder.

## 2. Case Presentation

A 44-year-old male originally from Nigeria and residing in the United States since his teenage years with a medical history of hypertension, significant alcohol use disorder, and no previous abdominal surgery who presented initially with altered mental status is associated with bilateral lower extremity weakness. These occurred while the patient was fasting and concurrently using hydrochlorothiazide to lose weight. Initial vital signs were blood pressure 163/106 mmHg, heart rate 113 beats per minute, respiratory rate 16 breaths per minute, temperature 36.9°C, and oxygen saturation 99%. Laboratory tests showed a white blood cell count of 15,900 with no bandemia, hemoglobin 9.7 g/dL, platelet count 379,000, sodium 133 mEq/L, potassium 2.3 mEq/L, magnesium 2.4 mEq/L, blood urea nitrogen 6 mg/dL, and creatinine 0.75 mg/dL. Glucose was 153 mg/dL, lactate 1.6 mmol/L, aspartate aminotransferase 66 U/L, alanine aminotransferase 43 U/L, total bilirubin 2.33 mg/dL, alkaline phosphatase 123 U/L, and albumin 3.4 g/dL. Influenza and COVID-19 tests were negative, and blood cultures from two sets showed no growth.

Urinalysis showed white blood cells less than 5 per high power field, protein 1+, blood 3+, and urine red blood cells 10–20 per high power field. Urine sodium was 18 mEq/L, urine potassium 34 mEq/L, urine chloride 64 mEq/L, urine osmolality 556 mOsm/kg, and serum osmolality 319 mOsm/kg. A transtubular potassium gradient greater than seven indicated renal potassium wasting. Aldosterone levels were less than 1 ng/dL, excluding hyperaldosteronism.

Computed tomography (CT) and head magnetic resonance imaging (MRI) revealed no acute process. Lumbar puncture showed cerebrospinal fluid with nucleated cells 2 per microliter, red blood cells 47 per microliter, glucose 76 mg/dL, and protein 79 mg/dL. Cerebrospinal fluid varicella-zoster virus and herpes simplex virus polymerase chain reaction tests were negative, and cerebrospinal fluid cultures showed no growth. Tests for anti-N-methyl-D-aspartate receptor (NMDAR) antibody and syphilis enzyme immunoassay were negative. These findings led to a diagnosis of metabolic encephalopathy due to hypokalemia and severe dehydration. The patient was admitted to the medical floor for aggressive fluid and potassium resuscitation, and for blood pressure management, lisinopril was started. A psychiatric evaluation revealed delirium secondary to electrolyte imbalances, and chlordiazepoxide (Librium) 25 mg three times daily was administered for alcohol withdrawal prophylaxis due to the patient's history of alcohol use. A therapeutic dose of intravenous (IV) thiamine was given due to suspected Wernicke's Korsakoff syndrome, but the response was not significant. Haloperidol was initiated to manage acute psychosis and agitation secondary to delirium.

As time progressed, the patient's abdomen exhibited progressive distension, accompanied by worsening nausea, vomiting, and diffuse, heaviness-like abdominal pain. Persistent hypokalemia continued despite aggressive replacement efforts. Notably, the patient not had a bowel movement for 5 days, and complete obstipation was evident without a previous history of constipation.

**Figure 1:**
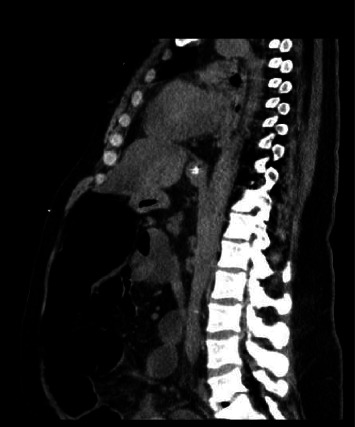
CT scan of the abdomen showing diffuse dilatation of the colon without evidence of obstruction.

Physical examination revealed a distended abdomen without tenderness, a tympanic note on percussion, and no signs of an acute abdomen. Bowel sounds were sluggish, and the patient declined a digital rectal examination. A CT scan of the abdomen with IV and rectal contrast depicted a diffuse dilatation of the colon with no evidence of an obstructing lesion (Figure 1). The maximum transverse dimension of the colon is approximately 8.4 cm without signs of ischemia or perforation. Surgical consultation yielded no planned intervention, and nasogastric and rectal tubes were inserted. Unfortunately, the patient's hemodynamic instability necessitated pressor support, leading to a transfer to the intensive care unit (ICU). Empiric antibiotics were initiated for suspected ischemic colitis, and due to the patient's inability to tolerate enteral feeding, total parenteral nutrition (TPN) commenced. Blood cultures, urine analysis, chest x-ray, and Trypanosoma serology returned negative results. A subsequent colonoscopy revealed diverticulosis coli, markedly edematous mucosa, distended colon without evidence of ischemic colitis, and no signs of pseudomembranous colitis or obstruction. Decompression of the colon through suction irrigation notably improved the patient's condition, allowing for the tolerance of oral feeds. Antibiotics were discontinued due to repeated negative blood cultures and unremarkable workups. His transient shock status was attributed to a combination of hypovolemia and medication side effects.

**Figure 2:**
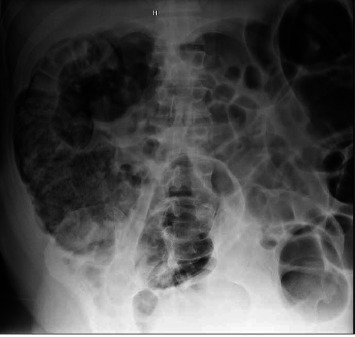
Plain abdomen x-ray showing proximal colonic dilatation.

Despite this initial improvement, the patient's condition deteriorated a few days later, characterized by recurrent abdominal distention without pain, persistent nausea and vomiting, and poor oral intake. A physical exam revealed a distended abdomen with generalized tenderness, no grading or rigidity, and present bowel sounds. Imaging revealed proximal colonic dilatation (Figure 2) with a cecal diameter of 10 cm. Erythromycin was trialed without success, prompting a return to the ICU, where TPN was reinserted. Rectal and nasogastric tubes proved insufficient for decompression, leading to the administration of neostigmine 2 mg IV twice. Remarkably, this intervention significantly improved the patient's condition, and the abdomen's distention was significantly reduced and softened with normal bowel sounds and mild tenderness. Subsequent abdominal x-rays confirmed the resolution of the dilatation and reduction of colonic diameter. Hypokalemia persisted despite aggressive repletion, even though magnesium levels were normal. Consequently, spironolactone was added, which led to an increase in potassium levels. Eventually, the patient's symptoms resolved, and he was discharged after a complete recovery.

## 3. Discussion

Ogilvie syndrome is an uncommon condition affecting approximately 0.1% of inpatient admissions annually [1]. The exact pathogenesis is still unclear, with numerous debated etiologies over the years. The association with trauma and spinal anesthesia might indicate a link to autonomic dysfunction [4]. Generally, Ogilvie syndrome is considered a diagnosis of exclusion, necessitating a comprehensive evaluation, including history, physical exam, labs, and imaging before a definitive diagnosis can be made. Clinically, this condition might resemble an ileus; key features to differentiate between the two include the presence or absence of bowel sounds and imaging findings. Ileus is typically characterized by the absence of bowel sounds and dilation of both the small and large bowels whereas Ogilvie syndrome usually presents with bowel sounds and mainly affects the colon, especially on the right side [5]. CT scan with oral and IV contrast is the preferred modality for diagnosis. Classically, Ogilvie syndrome on CT scan will show isolated dilatation of the cecum and ascending colon with a gradual transition zone or “cut off” at the splenic flexure. Importantly, diagnostic colonoscopy should be avoided in all patients with suspected Ogilvie syndrome, as gas insufflation is associated with an increased risk of perforation [6]^,^

Furthermore, assessment for potentially reversible causes of Ogilvie syndrome and attention to possible complications like ischemia or perforation is essential. Laboratory tests are usually nonspecific, but it might be helpful to assess for establishing the diagnosis. Elevated C-reactive protein (CRP), lactate, and leukocytosis raise concerns for colonic ischemia as an important possible complication of Ogilvie syndrome to be considered, especially in elderly or those with bowel ischemia risk factors. Testing for electrolytes, liver function tests, lipase, thyroid-stimulating hormone (TSH), and human chorionic gonadotropin (HCG) should be obtained to help rule out other causes of acute abdominal pain and detect conditions commonly associated with this condition, such as hypokalemia.

Early identification and prompt treatment of Ogilvie syndrome are crucial, irrespective of its underlying cause. This condition has the potential to progress rapidly, leading to necrosis or perforation, resulting in a more unfavorable prognosis and increased mortality rates. The primary approach involves conservative management, encompassing bowel rest and decompression through nasogastric and rectal tubes and addressing potential causative factors [7]. As a first-line treatment, pharmacological agents like neostigmine may be introduced if conservative measures prove ineffective or if the cecal diameter is more than 12 mm. However, neostigmine should be used in closed monitored settings only after excluding ischemia and perforation [4]. The use of erythromycin and other prokinetics is a matter of debate.

While colonoscopic decompression holds superior diagnostic value and plays a pivotal role in excluding alternative diagnoses, it is reserved for cases where pharmacological agents prove ineffective or contraindicated, primarily due to the elevated risk of recurrence and complications. Surgical interventions become a consideration in more intricate scenarios involving complications such as perforation, ischemia, or acute abdomen [4]. This stepwise approach ensures that less invasive measures are exhausted before resorting to more invasive procedures, thereby optimizing the balance between diagnostic precision and therapeutic efficacy.

The pathophysiology of Ogilvie syndrome is thought to involve autonomic dysfunction, with an increased activity of sympathetic activity in critically ill patients combined with decreased parasympathetic activity [8]. Temporary parasympathetic dysfunction in the sacral plexus is believed to result in atony of the distal large intestine, causing functional obstruction. Acetylcholine, the main neurotransmitter of the parasympathetic system, is broken down by acetylcholinesterase at the neuromuscular junction. Neostigmine, an acetylcholinesterase inhibitor, raises acetylcholine levels, thereby enhancing parasympathetic activity and improving contraction of colonic smooth muscle contractility [9]. However, that it might lead to bradycardia, hemodynamic instability, dizziness, salivation, nausea, vomiting, cramps, bronchospasm, headache, itching, and muscle weakness [9].

Our patient presented with recurrent Ogilvie syndrome at the atypical age of 46, along with multiple possible etiologies suggesting different underlying pathophysiologic pathways. Despite the association of many medications with this condition, discontinuation of haloperidol did not reverse the syndrome. In contrast, administering neostigmine led to clinical improvement, indicating that parasympathetic dysfunction might be a more likely cause. We also considered the possibility of chronic trypanosomiasis causing gastrointestinal ganglionic destruction over several decades, although its rarity makes this scenario less likely [10] as evident by negative Trypanosoma serology results. Chronic alcoholism and hypokalemia were identified as contributing factors, but the lack of improvement with electrolyte repletion suggests these were not the primary causes. Despite neostigmine being the first line of treatment, it could not be used due to its potential impact on blood pressure and concern of colonic ischemia. Therefore, a decompressive colonoscopy was performed, providing relief and highlighting the necessity of tailored treatment strategies in managing atypical presentations of Ogilvie syndrome. This case underscores the importance of recognizing Ogilvie syndrome in younger patients and navigating complex treatment challenges, thereby increasing awareness and understanding of this condition.

## 4. Conclusions

This case highlights the significance of identifying Ogilvie syndrome, particularly in atypical presentations and younger patients. It emphasizes also the necessity of addressing underlying causes and implementing tailored treatment strategies to effectively manage complex cases and prevent potential complications such as perforation or ischemia.

## Data Availability

The data used to support this study are included within the article. Further inquiries can be directed to the corresponding authors.
